# Proteogenomic Analysis on RNA m6A Modification-Associated Genes Identifies a Distinct Subgroup with High IGF2BPs Expression Across Cancer Types

**DOI:** 10.7150/ijms.115609

**Published:** 2025-08-16

**Authors:** Yebin Ryu, Eunhyong Chang, Hayoon Park, Sung-Yup Cho, Joon-Yong An

**Affiliations:** 1 Department of Integrated Biomedical and Life Science, Korea University, Seoul 02841, Republic of Korea.; 2 L-HOPE Program for Community-Based Total Learning Health Systems, Korea University, Seoul 02841, Republic of Korea.; 3 School of Biosystem and Biomedical Science, College of Health Science, Korea University, Seoul 02841, Republic of Korea.; 4 Department of Biomedical Sciences, Seoul National University College of Medicine, Seoul, Republic of Korea.; 5 Genomic Medicine Institute, Medical Research Center, Seoul National University, Seoul, Republic of Korea.; 6 Cancer Research Institute, Seoul National University, Seoul, Republic of Korea.

**Keywords:** m6A, IGF2BP, Pan-cancer, Multi-omics, Precision medicine, Cell cycle, Immune infiltration

## Abstract

**Background**: RNA N6-methyladenosine (m6A) modification is a key epitranscriptomic mechanism that regulates post-transcriptional gene expression. Although m6A-associated regulators have been implicated in cancer, their context-dependent roles and impacts on tumor heterogeneity remain incompletely defined.

**Methods**: We conducted a pan-cancer proteogenomic analysis of m6A-dependent mechanisms using multi-omics datasets from the Clinical Proteomic Tumor Analysis Consortium, utilizing genomic, transcriptomic, proteomic, and phosphoproteomic data. Unsupervised clustering based on expression of m6A regulatory genes identified distinct subgroups. We integrated m6A-seq and RIP-seq data from cancer cell lines and analyzed the immune deconvolution results to define m6A-driven regulatory programs and assess tumor immune infiltration across subgroups.

**Results**: Three molecular subgroups (IGF2BP-H, -M, and-L) were defined based on the expression patterns of m6A readers, with IGF2BP1/2/3 acting as the primary markers distinguishing the subgroups. Their upregulation has been attributed to either copy number amplification or transcription factor activation, depending on the tumor context. The IGF2BP-H subgroup exhibited enhanced cell cycle activity, which was supported by concordant transcriptomic, proteomic, and phosphoproteomic signatures. Mechanistic analyses revealed that IGF2BPs directly bind to and stabilize m6A-modified transcripts, including TOP2A, ANLN, and TFRC, thereby promoting their translation and contributing to cell cycle progression. IGF2BPs also enhanced VEGFA expression in head and neck squamous cell carcinoma and pancreatic ductal adenocarcinoma, potentially promoting immunosuppressive signaling. Immune deconvolution revealed reduced CD8^+^ T cell infiltration in IGF2BP-H tumors, suggesting a less inflamed microenvironment and potentially diminished responsiveness to immunotherapy.

**Conclusion**: Our results highlight the pivotal role of IGF2BP in governing m6A-dependent regulatory mechanisms in cancer cells, highlighting their potential link with aggressive tumor behavior and immune evasion. This study provides important insights into the heterogeneity of m6A-related processes across different malignancies and reveals potential avenues for therapeutic interventions.

## Introduction

N^6^-methyladenosine (m6A) is the most abundant internal modification of eukaryotic mRNA and influences the metabolism of a broad spectrum of RNA 1, 2. This dynamic epitranscriptomic mark governs key processes in gene expression, such as mRNA splicing, translation, stability, and translocation, ultimately shaping the functional output of the transcriptome 3-6. RNA m6A modification is initiated by a methyltransferase complex (writers), reversed by demethylases (erasers), and interpreted by m6A-binding proteins (readers) to mediate specific cellular functions 7, 8. In the context of cancer, dysregulated m6A-associated genes (writers, erasers, and readers) have been implicated in tumor initiation, progression, metastasis, altered metabolism, drug resistance, and immune evasion 9-12.

Despite the substantial progress in understanding the biological functions of m6A, the complexity of its regulatory networks in different cancer types remains unclear. m6A is a highly diverse, reversible modification that can be added to or removed from the same mRNA 13, 14. Although previous research has focused on individual cancer types in a target-specific manner 15, recent findings suggest that m6A methylation may facilitate or inhibit tumor progression depending on the cellular context. Because of the dynamic and complex methylation patterns of m6A, it remains difficult to precisely predict how the overall methylation patterns of m6A influence tumor progression in individual patients. Therefore, it is necessary to perform a comprehensive analysis of m6A-associated genes across multiple cancer types using actual patient data to gain a clearer understanding of how m6A contributes to tumor characteristics across different malignancies. Since m6A directly influences translation, thereby affecting protein abundance and phosphorylation status 16, 17, a multi-omics approach integrating transcriptome, proteome, and phosphoproteome data is needed to fully characterize how m6A modification ultimately affects the phenotype and its contribution to tumor progression 18. A pan-cancer analysis can further identify common m6A regulatory signatures across cancer types and provide insights for developing broadly applicable biomarkers and therapeutic interventions.

In this study, we performed a comprehensive analysis of 25 m6A-associated genes across ten different cancer types to elucidate how m6A regulatory patterns affect tumor subgroups. Our results showed that, the expression of m6A regulators was associated with subgroup differentiation in six of these cancer types, with the IGF2BP1/2/3 family emerging as a key factor in defining these subgroups. High IGF2BPs expression was correlated with enhanced cell cycle activity and reduced immune responses, which could be explained by its direct interaction with cell cycle-related target transcripts. IGF2BPs also shaped the tumor microenvironment by promoting immunosuppressive features and influencing responses to immunotherapy. By highlighting the pivotal influence of IGF2BPs on cancer progression, our findings advance the current understanding of m6A-dependent regulatory mechanisms and suggest potential therapeutic targets for diverse malignancies.

## Materials and Methods

### Data Collection and Preprocessing

We obtained genomic, transcriptomic, global proteomic, and phosphoproteomic data for ten cancer types, i.e., breast cancer (BRCA) 19, clear cell renal cell carcinoma (CCRCC) 20, colon adenocarcinoma (COAD) 21, glioblastoma (GBM) 22, high-grade serous carcinoma (HGSC) 23, head and neck squamous cell carcinoma (HNSCC) 24, lung squamous cell carcinoma (LSCC) 25, lung adenocarcinoma (LUAD) 26, pancreatic ductal adenocarcinoma (PDAC) 27, and uterine corpus endometrial carcinoma (UCEC) 28, from the Clinical Proteomic Tumor Analysis Consortium. For genomic data, mutation annotation (MAF) files and segment-level copy number variant (CNV) data were retrieved from the GDC data portal (https://portal.gdc.cancer.gov). Transcriptomic data were downloaded in transcripts per million format and subjected to k-nearest neighbor imputation with k = 5 for genes with less than 30% missing values. Subsequently, the data were log2-transformed and quantile-normalized across the samples. Proteomic data preprocessed by the Broad Institute were downloaded from the PDC data portal (https://proteomic.datacommons.cancer.gov/pdc/) and imputed using the same strategy used for transcriptomic data. Phosphoproteomic data were obtained from LinkedOmics (https://www.linkedomics.org/) and normalized using z-scores to standardize global phosphoprotein levels across various cancer types.

### Clustering by m6A-associated Genes

We reviewed the literature on m6A modifications and selected 25 m6A regulators to identify distinct patterns of m6A modification 29-31. The 25 m6A regulators included 10 writers (METTL3, METTL5, METTL14, METTL16, WTAP, RBM15, RBM15B, CBLL1, ZC3H13, and KIAA1429), 2 erasers (FTO and ALKBH5), and 13 readers (YTHDF1, YTHDF2, YTHDF3, YTHDC1, YTHDC2, HNRNPC, HNRNPA2B1, IGF2BP1, IGF2BP2, IGF2BP3, FMR1, ELAVL1, and LRPPRC). We used Uniform Manifold Approximation and Projection (UMAP) for dimensionality reduction based on the expression profiles of these 25 m6A regulators using the umap R package (v0.2.10). The data were subsequently clustered using k-means clustering. We calculated the mean silhouette scores for different values of k to optimize the clustering process. Cancer types with a maximum silhouette score >0.6 were selected for further analysis.

### Survival Analysis

Overall survival (OS) analysis was performed to assess the prognostic significance of IGF2BP-based molecular subgroups. Clinical metadata, including OS time and OS event status, were integrated with m6A subgroup data, and samples from the IGF2BP-H and IGF2BP-L groups were selected for survival comparison. Kaplan-Meier survival curves were generated using the survfit() function from the survival R package (v3.7.0) based on survival objects constructed with the Surv() function. Differences in survival between the IGF2BP-H and -L groups were evaluated using log-rank tests. The statistical significance of the survival difference was assessed and visualized using the ggsurvplot() function in the Survminer R package (v0.4.9).

### Copy Number Variation (CNV) Analysis

Gene-level CNV data for multiple cancer types were obtained from the PDC data portal and processed by the Washington University team. CNV values were categorized into three discrete levels—Loss, Neutral, and Gain—based on cutoff values of < -0.2, between -0.2 and 0.2, and >0.2, respectively. For downstream analyses, only gain events were retained to evaluate oncogenic amplification. Each CNV dataset was transformed into the long format and merged with the m6A subgroups. We integrated CNV annotations with mutation data (MAF) using the maftools R package (v2.18.0) 32 to assess the association between the m6A subgroup and copy number amplification of IGF2BP1, IGF2BP2, and IGF2BP3. Fisher's exact test was used to compare the amplification frequencies of the three IGF2BPs genes between the IGF2BP-H and -L subgroups for each cancer type using the mafCompare() function.

### Differential Expression Analysis

For the differential expression analysis, raw RNA count data were processed using the DESeq2 package in R (v1.42.1) 33. Gene expression counts were extracted and transformed into a count matrix with genes as rows and samples as columns. The count matrix is a subset that retains only the samples present in the clinical metadata. The variance-stabilizing transformation was applied to normalize the count data, and genes with an average read count < 50 were excluded. A DESeq DataSet was created using cluster classification as the design variable, and a differential expression analysis was performed using the DESeq function with parallel computing. The results, including log2 fold changes (log2FC) and adjusted *p*-values, were extracted. Genes with a false discovery rate (FDR) below 5% were considered significant.

### Transcription Factor Activity Estimation

We used the decoupleR 34 R package, leveraging the CollecTRI 35 TF-target database, to estimate transcription factor (TF) activity. Differentially expressed genes (DEGs) were obtained, and log2FC was used as input for the universal linear model approach via the run_ulm function to compute the TF activity scores. A statistical model was built using *t*-statistics from the DEGs as inputs and the CollecTRI network as prior knowledge, specifying source nodes as TFs and target nodes as regulated genes. Significantly enriched TFs were selected using an FDR threshold of 0.1.

### Gene Set Enrichment Analysis (GSEA)

We conducted GSEA 36 and gene set variation analysis (GSVA) 37 to explore the underlying biological pathways associated with transcriptomic, proteomic, and phosphoproteomic data. For GSEA, we compared the three groups (IGF2BP-H, -M, and -L) by performing a DEG analysis. Various databases, including Gene Ontology (GO) biological processes, Kyoto Encyclopedia of Genes and Genomes (KEGG) pathways, and Reactome genes, were used, with a significance threshold of an FDR-adjusted *p*-value < 0.05. We also applied GSVA, utilizing the “zscore” method from the GSVA R package, to assess pathway activity at the sample level using global proteome and phospho-proteome data tested with Hallmark gene sets. Following pathway activity scoring, we conducted *t*-tests between the IGF2BP-H and -L groups to identify the significantly different pathways.

### Protein-protein Interaction (PPI) Enrichment Analysis

PPI enrichment analysis was performed using Metascape 38, a web-based platform. Differentially expressed proteins (DEPs) with a log2FC > 1 were selected from each cancer type and used as input for Metascape to identify key protein interactions in the IGF2BP-H condition. For a comprehensive assessment, a PPI network analysis was conducted using a combined dataset of six cancer types. The interactome network was constructed using the Metascape integrated knowledge base, incorporating high-confidence PPIs. A complex molecular detection (MCODE) algorithm was applied to extract densely connected subnetworks and identify biologically relevant modules. Each MCODE component was functionally annotated based on the three most significantly enriched pathways.

### Collection and Analysis of IGF2BPs-associated Sequencing Data

For the collection and analysis of IGF2BPs RIP-seq and m6A-seq data, we used publicly available datasets generated by Huang et al. (2018) 39 and Shwartz et al. (2014) 40, which are accessible through the GEO database. We analyzed RIP-seq data for IGF2BP1, IGF2BP2, and IGF2BP3 in the HepG2 cell line (GSE90639) and m6A-seq data (GSE90642 and GSE55572). Raw FASTQ files were aligned to the human reference genome (GRCh38) using the HISAT2 aligner. The aligned BAM files were used to generate coverage plots using the KaryoploteR (v1.28.0) R package 41. Additionally, we examined the RNA-seq data from HepG2 cells subjected to shRNA-mediated knockdown of IGF2BP1, IGF2BP2, and IGF2BP3 (GSE90684). Raw count data were normalized using between-sample quantile normalization, and data from replicates 1 and 2 treated with shRNA for one hour, were used for further analysis. Differential expression analysis was performed using the DESeq2 package to determine the significance of the changes in gene expression levels.

### Kinase Library Enrichment Analysis

The Kinase Library is a database that experimentally characterizes the substrate motif specificity of 303 Ser/Thr protein kinases and 78 Tyr protein kinases 42, 43. In this study, phosphorylation site motifs were scored based on their similarity to the preferred motifs of 303 Ser/Thr and 78 Tyr protein kinases using percentile scores. Each unique singly phosphorylated site was assigned to a biochemically predicted kinase if it ranked within the top 15 Ser/Thr kinases or within the top eight Tyr kinases, based on these scores. For motif-based enrichment analysis, phosphorylation sites with log2FC > 0.5 and FDR < 0.05 were set as the foreground dataset, while all other phosphorylation sites were used as the background dataset. The log₂ frequency factor for each kinase was calculated by comparing the proportion of biochemically favored phosphorylation sites for that kinase in the foreground and background sets. The Haldane correction was applied by adding a count of 0.5 to correct for zero-frequency cases. Statistical significance was assessed using a one-sided Fisher's exact test, and *p*-values were adjusted using the Benjamini-Hochberg method.

### Immunotherapy Response Prediction Using Tumor Immune Dysfunction and Exclusion (TIDE)

We used a TIDE computational framework to predict the response of NSCLC samples to immunotherapy 44. Bulk RNA-seq data from LSCC and LUAD samples were input into the TIDE web tool (http://tide.dfci.harvard.edu/), which predicts the response to immune checkpoint blockade (ICB) based on two mechanisms of tumor immune evasion: T-cell dysfunction and T-cell exclusion. For each cancer type, tumors were classified as either responders or non-responders based on the TIDE prediction score. We counted the number of true-positive (responders) and false-positive (non-responders) samples in each group for both LSCC and LUAD, and Fisher's exact test was used to determine if the differences in immunotherapy response between these groups were statistically significant.

### Use of Generative AI in the Writing Process

During manuscript preparation, OpenAI's ChatGPT-4o was used to improve the clarity and readability of the text. Following the use of this tool, all content was thoroughly reviewed and revised by the authors, who take full responsibility for the final version of the manuscript.

## Results

### IGF2BPs Highlight a Distinct m6A Subgroup Across Six Cancer Types

We investigated 25 m6A-associated genes to identify subgroups with distinct m6A modification patterns that exhibit dynamic regulation within cells. We used transcriptomic, proteomic, and phosphorproteomic datasets from 1,060 patients with 10 cancer types: BRCA, ccRCC, COAD, GBM, HGSC, HNSCC, LSCC, LUAD, PDAC, and UCEC.

**Figure 1:**
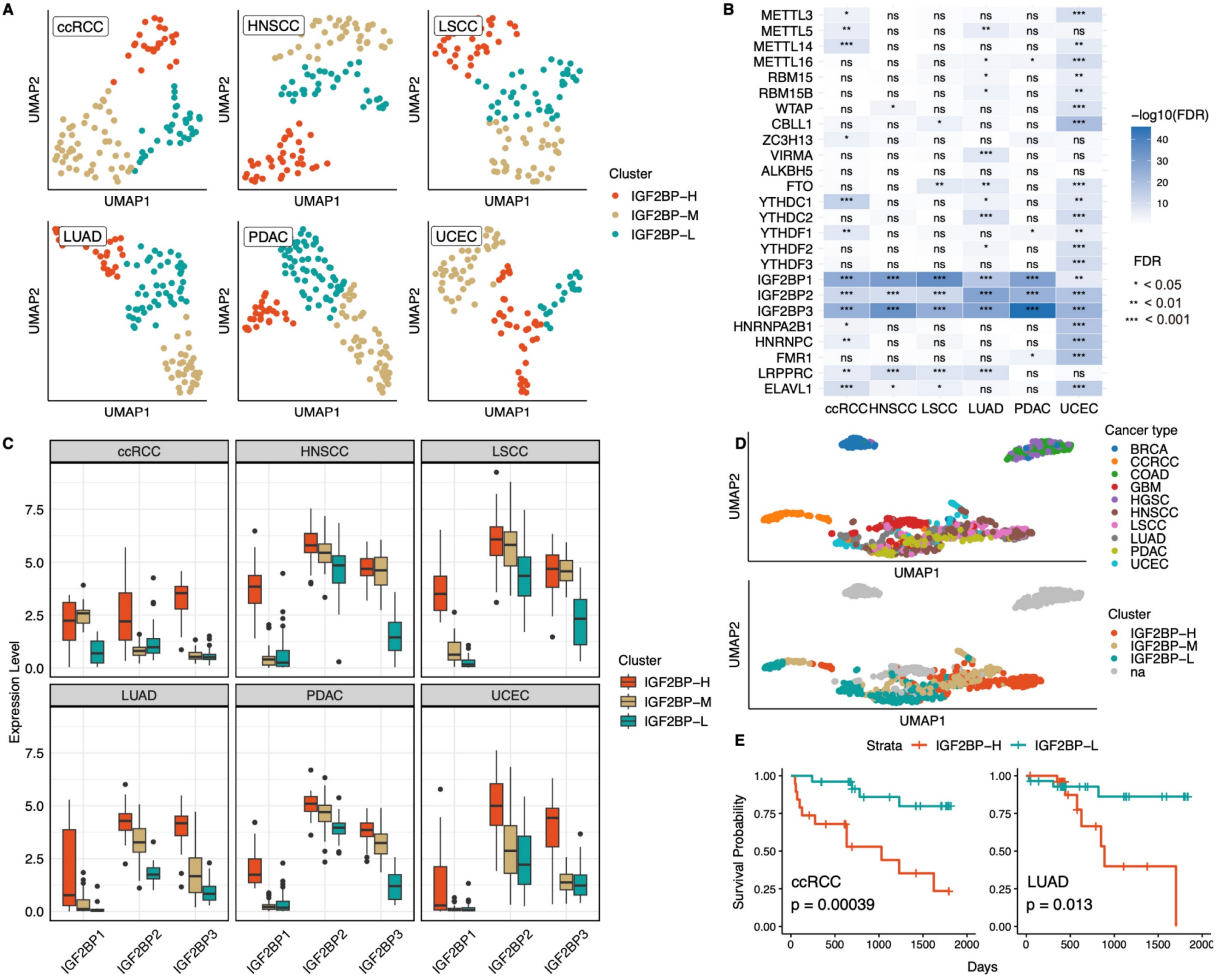
** Identification of m6A regulatory pattern in 10 cancer types. A:** UMAP clustering of samples from six cancer types (ccRCC, HNSCC, LSCC, LUAD, PDAC, and UCEC), based on the expression of 25 m6A-associated genes. Samples were grouped into three distinct clusters for each cancer type. ccRCC, Clear cell renal cell carcinoma; HNSCC, Head and neck squamous cell carcinoma; LSCC, Lung squamous cell carcinoma; LUAD, Lung adenocarcinoma; PDAC, Pancreatic ductal adenocarcinoma; UCEC, Uterine corpus endometrial carcinoma. **B:** Heatmap showing the statistical significance of differential expression of m6A-associated genes between clusters in each cancer type. IGF2BP1, IGF2BP2, and IGF2BP3 consistently served as the most significant regulators, distinguishing clusters across all six cancer types. Statistical significance was assessed using the Kruskal-Wallis test, followed by FDR correction. ns indicates non-significance. **C:** Boxplots illustrating the expression levels of IGF2BP1, IGF2BP2, and IGF2BP3 across the three clusters (IGF2BP-H, IGF2BP-M, and IGF2BP-L) in each cancer type. **D:** UMAP visualization of all cancer samples based on the expression of the 25 m6A-associated genes. Among the six cancer types that formed distinct clusters, five (excluding ccRCC) were grouped. na represents cancer types not included in the six cancer types. **E:** Kaplan-Meier survival analysis comparing the IGF2BP-H and -L groups in ccRCC (left) and LUAD (right). Significant differences in survival probability were observed between both cancer types (ccRCC, *p* = 0.00039; LUAD, *p* = 0.013), indicating the potential prognostic role of IGF2BPs.

When conducting transcriptome-based clustering of each cancer type with 25 m6A-associated genes, only six cancer types (ccRCC, HNSCC, LSCC, LUAD, PDAC, and UCEC) were clustered with silhouette scores >0.6 (**Fig. 1A, Table S1A, S1B**). This suggested that the samples from these six cancer types can be distinguished by their distinct m6A modification patterns and grouped among the cancer types. Moreover, when we performed proteomics-based clustering of the same samples into three groups, patients exhibited clustering patterns similar to those identified by the transcriptomics-based approach for all cancer types except ccRCC (**Fig. S1A**). These findings implied that the distinct m6A modification signatures identified at the transcript level are consistently reflected at the protein level, underscoring the robustness of these m6A-associated patterns across different molecular layers.

The expression levels of m6A-associated genes varied significantly between clusters, with the IGF2BP family exhibiting the most pronounced differences among subgroups across all six cancer types and demonstrating the highest feature importance among the 25 examined genes in the clustering analysis (**Fig. 1B, S1B**). Examination of IGF2BPs expression across each cluster further revealed that IGF2BP1, IGF2BP2, and IGF2BP3 consistently displayed similar patterns, with all three transcripts being the most highly expressed in one cluster, showing intermediate expression in another, and the lowest expression in the third (**Fig. 1C**). Consistent with these findings, proteomics-based clustering stratified IGF2BPs expression into high-, intermediate-, and low-expression groups, a pattern that was consistent across both the transcriptomic and proteomic levels (**Fig. S1C**). Based on these consistent patterns, we subsequently named the clusters as IGF2BP high (IGF2BP-H), middle (IGF2BP-M), and low (IGF2BP-L) groups, considering the overall expression profiles of IGF2BP1,2 and 3. We further confirmed this classification by calculating the average expression of IGF2BP1, IGF2BP2, and IGF2BP3 across cancer types and clusters, which consistently showed the highest levels in IGF2BP-H, intermediate in IGF2BP-M, and lowest in IGF2BP-L (**Table S1C**).

Furthermore, when projecting all cancer samples onto UMAP, we observed that among the six cancer types that formed distinct m6A-based clusters, five (excluding ccRCC) were clustered. Each of the IGF2BP-H, -M, and -L groups formed distinct clusters, irrespective of the cancer type (**Fig. 1D**), indicating that the IGF2BPs clusters shared common molecular characteristics among these five cancer types. When comparing survival rates between the IGF2BP-H and IGF2BP-L groups, significant differences were observed in ccRCC and LUAD (ccRCC, *p* = 3.9 × 10⁻⁴; LUAD, *p* = 0.013), whereas no significant differences were found in the other cancer types (**Fig. 1E**). When all three subgroups were compared, the IGF2BP-M group exhibited intermediate survival in ccRCC and LUAD, whereas survival differences among the subgroups remained non-significant in the other cancer types (**Fig S1D**). These findings highlighted the emergence of a distinct IGF2BP-driven m6A subtype across multiple cancer types, where elevated IGF2BPs expression correlated with poorer outcomes in some cases.

### Gene Amplification and Transcription Factor-mediated Upregulation of IGF2BPs in Six Cancer Types

**Figure 2:**
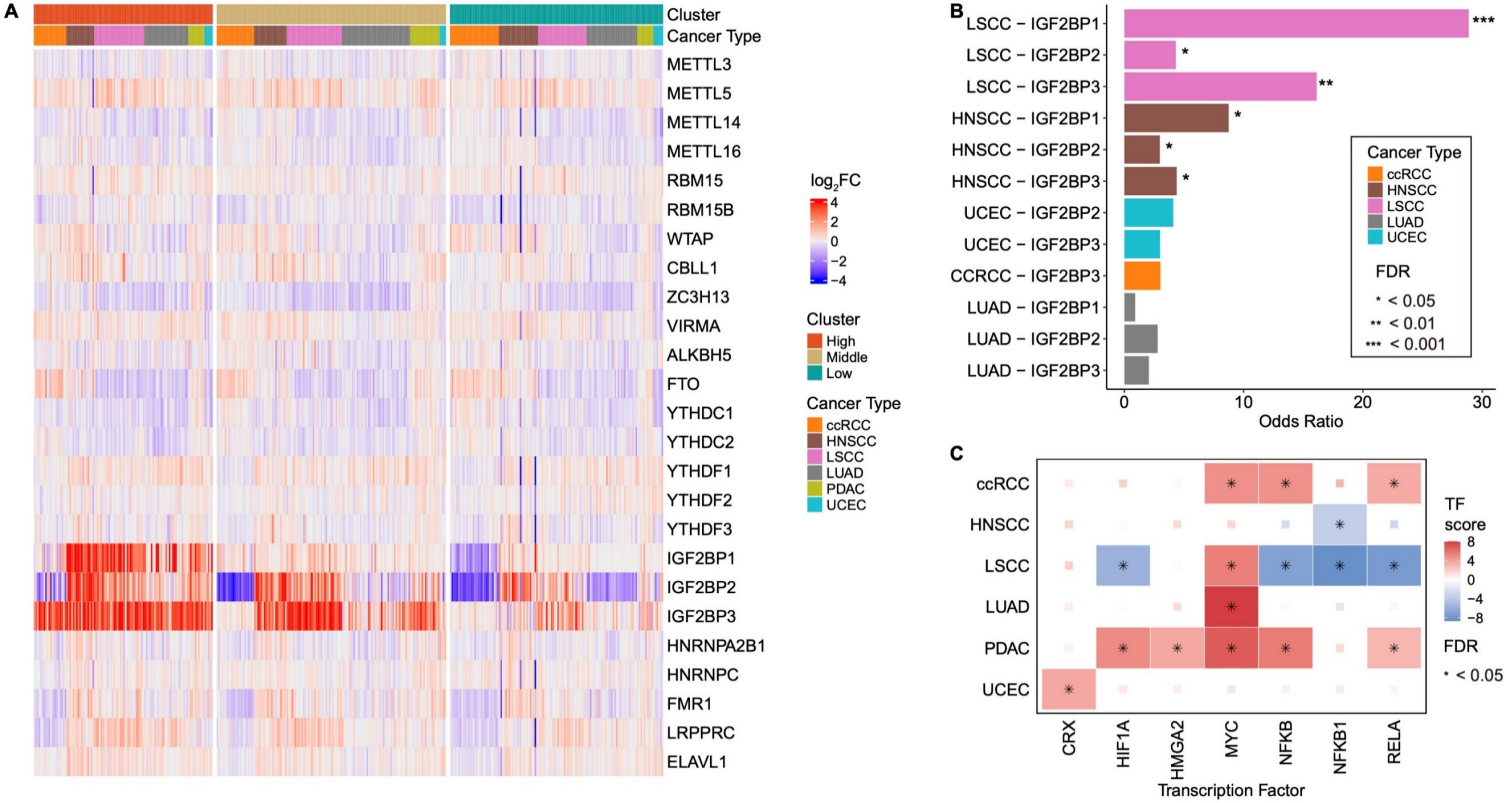
** Overexpression of IGF2BPs through copy number amplification and TF activation**. **A:** Heatmap showing the differential expression of 25 m6A-associated genes between tumor and normal tissues across six cancer types. IGF2BP1, IGF2BP2, and IGF2BP3 exhibited the most significant differences in their expression levels, with a clear separation between the IGF2BP-H, -M, and -L groups. **B:** CNV analysis comparing IGF2BP-H and -L groups. Significant copy number gain was observed in LSCC and HNSCC, particularly in IGF2BP1 and IGF2BP3. Statistical significance was assessed using Fisher's exact test with FDR correction. **C:** Analysis of TF activity in IGF2BPs-related regulatory networks across different cancer types. TF activity was significantly enriched in ccRCC, LUAD, PDAC, and UCEC, indicating potential regulatory roles of IGF2BPs in these tumor types. CNV copy number variation, TF transcription factor.

We analyzed the expression levels of 25 regulatory genes across six cancer types and compared them with the corresponding normal tissue samples to investigate dysregulated m6A-associated gene expression in tumors compared to normal tissues in each group. Notably, IGF2BP1, IGF2BP2, and IGF2BP3 exhibited the most pronounced differences (**Fig. 2A, Table S2A**). In the IGF2BP-H group, all three IGF2BPs were distinctly upregulated compared with those in normal cells, whereas their expression levels in the IGF2BP-L group were comparable to or lower than those in normal cells. Previous studies have shown that IGF2BP1, IGF2BP2, and IGF2BP3 exhibit peak expression levels during specific stages of embryonic development, with only IGF2BP2 remaining active in adult tissues. However, abnormal expression of IGF2BP2 and reactivation of IGF2BP1 and IGF2BP3 are frequently observed during cancer progression 45. Therefore, the significant upregulation of IGF2BP family members in the IGF2BP-H group suggested a potential link between tumorigenesis and cancer progression.

We next investigated the cause of the elevated expression of IGF2BPs and hypothesized that this could be attributed to two possible factors: gene amplification and TF activation. CNV gains of IGF2BP1, 2, and 3 were significantly more prevalent in IGF2BP-H group than in IGF2BP-L group in both LSCC and HNSCC (LSCC, adjusted *p* < 0.05; HNSCC, adjusted *p* < 0.1) (**Fig. 2B, Table S2B**). These findings indicated that the higher expression levels of IGF2BPs in LSCC and HNSCC are likely due to gene amplification, leading to an increased number of copies of these genes, thereby boosting their expression. TF activation also plays a crucial role in the elevated expression of IGF2BPs. According to the TF-target database, TFs associated with IGF2BPs include CRX, HIF1A, HMG2, MYC, NFKB, NFKB1, and RELA 35. We observed significant activation of several TFs in ccRCC and PDAC (ccRCC, FDR < 0.08; PDAC, FDR < 0.03) by estimating the activity of these TFs based on transcriptome data. Additionally, LUAD and UCEC showed the activation of one transcription factor each, MYC for LUAD and CRX for UCEC (LUAD, FDR = 1.3 x 10^-13^; UCEC, FDR = 0.027) (**Fig. 2C, Table S2C**). Therefore, while LSCC and HNSCC appear to experience increased IGF2BP1, 2, and 3 expressions due to gene amplification, ccRCC, LUAD, PDAC, and UCEC are likely to experience higher expression levels through the activation of transcription factors.

### Cell Cycle Pathways Are Upregulated in the IGF2BP-H Group

**Figure 3:**
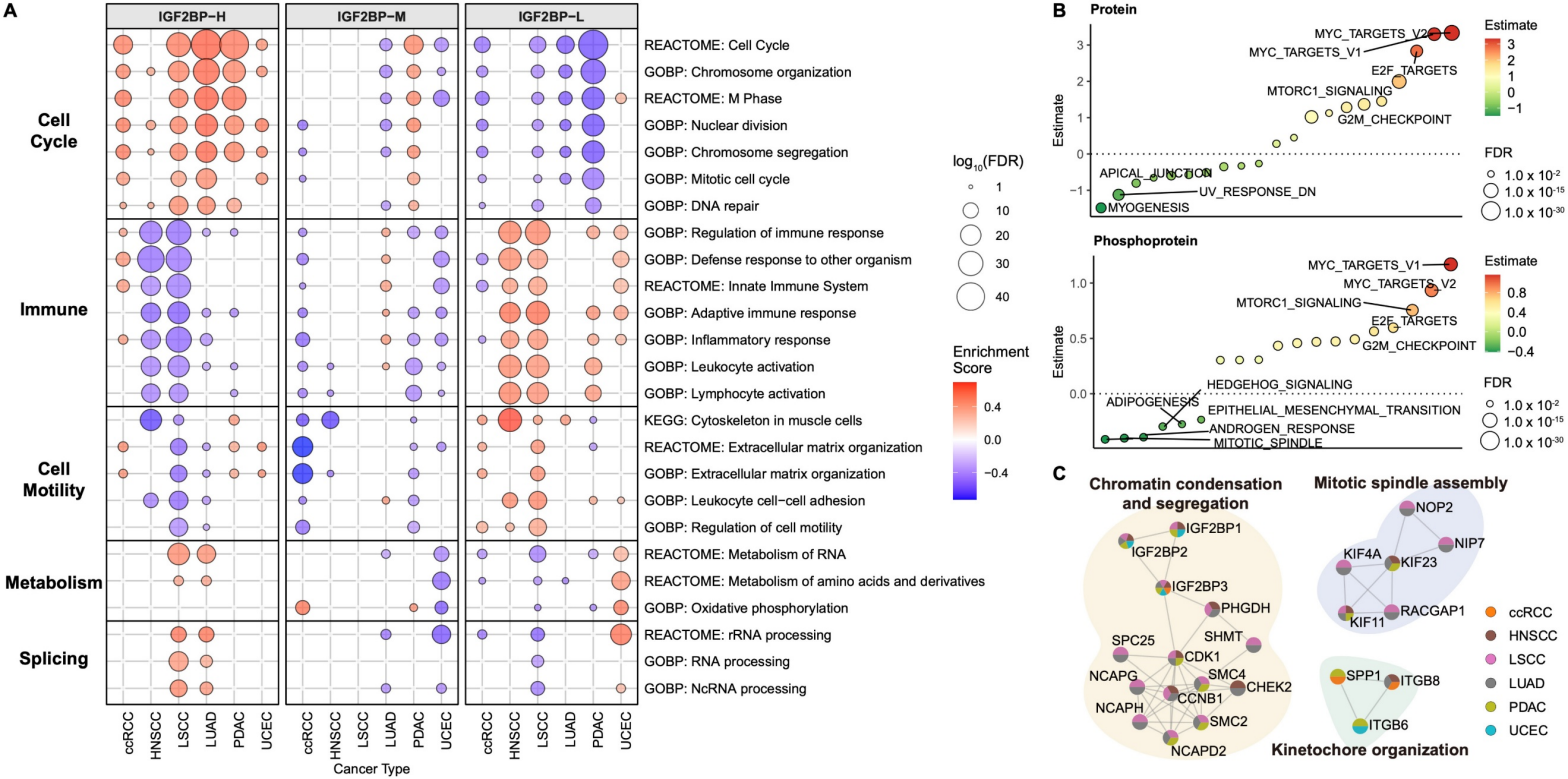
** Enriched pathways in IGF2BPs groups. A:** Bubble plot showing enriched biological pathways in the IGF2BP-H, -M, and -L groups across six cancer types. Bubble size represents the significance level (-log10 FDR) and color indicates the enrichment score. **B:** Single-sample gene set enrichment analysis comparing protein and phosphoprotein expression between the IGF2BP-H and -L groups, highlighting their potential roles in tumor progression. *P*-values were calculated using Welch's *t*-test and adjusted with FDR. **C:** Functional protein-protein interaction enrichment analysis of significantly upregulated proteins in the IGF2BP-H group. Each node represents a protein identified as significantly upregulated in the IGF2BP-H group. The colors within the pie chart indicate the cancer types (ccRCC, HNSCC, LSCC, LUAD, PDAC, UCEC) in which the protein was detected as a differentially expressed protein.

We then sought to identify the key pathways associated with each group based on transcriptomic data (**Fig. 3A, Table S3A**). In the IGF2BP-H group, cell cycle-related pathways were significantly upregulated across all six cancer types examined, indicating consistent enhancement of cell proliferation mechanisms. In contrast, the IGF2BP-L group predominantly exhibited elevated immune response pathways, suggesting heightened immune activity in these samples. The cell motility pathways were also more active in the IGF2BP-L group. In both LSCC and LUAD, the IGF2BP-H group showed increased activation of metabolic- and splicing-related pathways. The IGF2BP-M group generally exhibited characteristics intermediate between those of the IGF2BP-H and -L groups. Consequently, subsequent analyses focused primarily on comparisons between the IGF2BP-H and -L groups.

Consistent with these results, a comparison of IGF2BP-H and IGF2BP-L across the six cancer types using proteomic (**Table S3B**) and phosphoproteomic (**Table S3C**) expression data revealed the enrichment of cell cycle-related terms (**Fig. 3B**). These findings demonstrated a significant enrichment of cell cycle-related terms across multiple molecular layers in the IGF2BP-H group compared to the IGF2BP-L group. LUAD exhibited the most pronounced enrichment, whereas UCEC showed minimal differences between the IGF2BP-H and -L groups (**Fig. S2A, Table S3D**).

PPI enrichment analysis also showed cell cycle-related networks were upregulated in the IGF2BP-H group (**Fig. 3C, S2B, Table S3E**). Proteins interacting with IGF2BP1, 2, and 3 were predominantly enriched in pathways related to chromatin condensation and segregation. Additionally, the KIF protein family, as well as RACGAP1, NIP7, and NOP2 were significantly enriched in pathways critical for mitotic spindle assembly. Moreover, proteins such as SPP1, ITGB6, and ITGB8 were notably enriched in the pathways involved in kinetochore organization. The enhanced functionality of these protein interactions in the IGF2BP-H group facilitated active cell division, contributing to the observed proliferative characteristics.

### IGF2BPs Regulate Cell Cycle by Targeting TOP2A, ANLN, and TFRC

**Figure 4:**
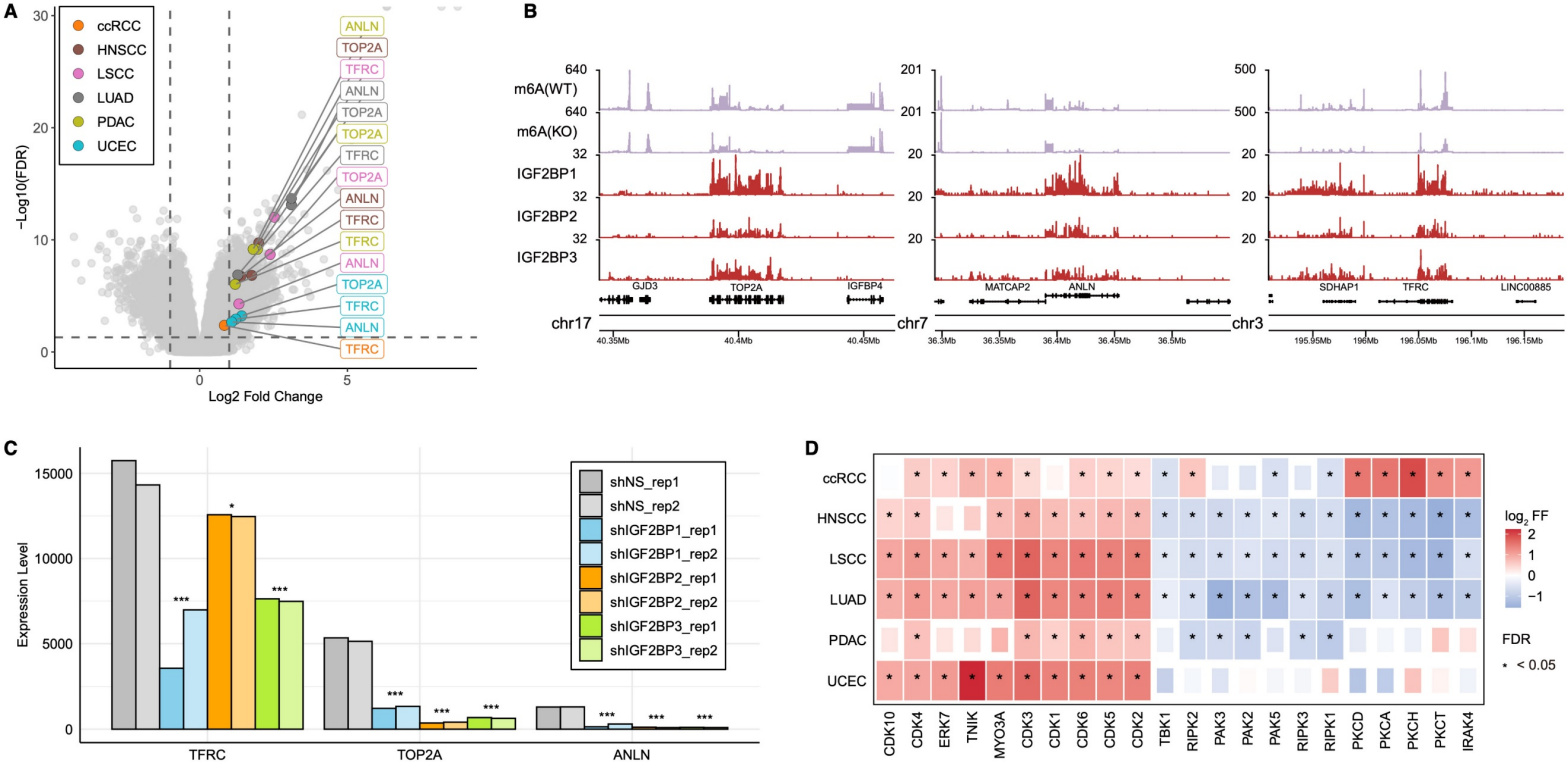
** IGF2BPs mediated regulatory interactions and downstream kinase activity shifts. A:** Volcano plot highlighting ANLN, TFRC, and TOP2A as consistently upregulated proteins in the IGF2BP-H group across the six cancer types. **B:** Coverage plots from m6A-seq and RIP-seq data in HepG2 cells. m6A(WT) and m6A(KO) represent m6A peak coverage in wild-type and m6A writer knockout conditions, respectively. IGF2BP1, IGF2BP2, and IGF2BP3 tracks show RIP-seq read coverage, indicating binding of each protein to the respective transcripts (TOP2A, ANLN, and TFRC). WT wild type, KO knock out. **C:** Gene expression changes in TFRC, TOP2A, and ANLN upon silencing of IGF2BP1, IGF2BP2, and IGF2BP3. *FDR < 0.05, **FDR < 0.01, ***FDR < 0.001. **D:** Differences in kinase activity predicted from phosphoproteomic analysis between the IGF2BP-H and -L groups, with significant changes marked by asterisks.

IGF2BPs function as m6A readers, bind to cell cycle-related genes, enhance their stability, and promote their translation 39, 46. We examined the highly expressed proteins in the IGF2BP-H group to gain more detailed insights into how IGF2BPs regulate the cell cycle within the IGF2BP-H group. Proteins, including TOP2A, BPI, PSAT1, ANLN, and TFRC, were upregulated in five of the six cancer types (**Fig. 4A, Table S4A**). Of these, TOP2A, ANLN, and TFRC are well-known for their roles in cell cycle regulation. Specifically, TOP2A encodes a DNA topoisomerase that relaxes double-helical DNA and is critical for chromosome segregation 47, ANLN is involved in cytokinesis 48, and TFRC regulates iron metabolism that is essential for cell growth 49. These three genes were also upregulated at the transcriptional level (**Fig. S3A**), and further pathway analysis showed that the subgroup of patients with high expression of these genes manifested activated cell cycle-related pathways, supporting the idea that these genes play key roles in cell cycling (**Fig. S3B, Table S4B**). These findings suggested that TOP2A, ANLN, and TFRC are candidate IGF2BPs targets that drive cell cycle progression in IGF2BP-H group.

In the HepG2 cell line, we confirmed that the transcripts of TOP2A, ANLN, and TFRC are marked by m6A methylation and bound by IGF2BP1/2/3, as revealed through the integration of RIP-seq and m6A-seq datasets (**Fig. 4B**). Data from the m6A-Atlas 50 further support the presence of m6A modifications in all three transcripts across several independent datasets (**Fig. S3C**). Upon silencing of IGF2BP1/2/3 in HepG2 cells, we observed a significant reduction in the abundance of these targets—TOP2A (FDR < 2.0 × 10^-34^), ANLN (FDR < 1.0 × 10^-12^), and TFRC (FDR < 0.017)—indicating that IGF2BP proteins contribute to the stability and/or translation of these transcripts (**Fig. 4C**, **Table S4C**). To assess whether these effects depend on m6A methylation, we re-analyzed RNA-seq datasets from multiple cancer cell lines under m6A writer silencing conditions 40, 51, 52. In most cases, knockout of these writers led to significant downregulation of the IGF2BPs targets, underscoring a functional dependency on m6A modification for their expression (**Fig. S3D, Table S4D**).

Further insights into the mechanistic underpinnings of enhanced cell cycle activity were obtained by examining phosphoprotein and kinase profiles (**Fig. 4D, Table S4E**). Estimation of kinase activity based on differentially expressed phosphoproteins revealed that the IGF2BP-H group exhibited significant activation of cyclin-dependent kinases (CDKs), which are pivotal in regulating the cell cycle 53. Additionally, kinases within the MAPK pathway, such as ERK7 54 and, as well as TNIK 55, which can activate the Wnt/β-catenin signaling pathway, and MYO3A 56, involved in cytoskeletal regulation and cell motility, were activated. Conversely, kinases that regulate inflammation and immune responses, including PKCA, PKCD, PKCH, PKCT, IRAK4, RIPK1, RIPK2, RIPK3, and TBK1^57^-^59^, were downregulated in at least three cancer types within IGF2BP-H group, with the exception of ccRCC, in which immune pathways were activated, consistent with our pathway enrichment results (**Fig. 3A**). Moreover, kinases that influence cytoskeletal dynamics and cell motility, such as PAK2, PAK3, and PAK5^60^, were similarly downregulated in IGF2BP-H group, further supporting our pathway enrichment findings regarding cellular structural and motility changes (**Fig. 3A**).

### m6A Subgroup Dependent Immune Landscape and Immunotherapy Resistance

**Figure 5:**
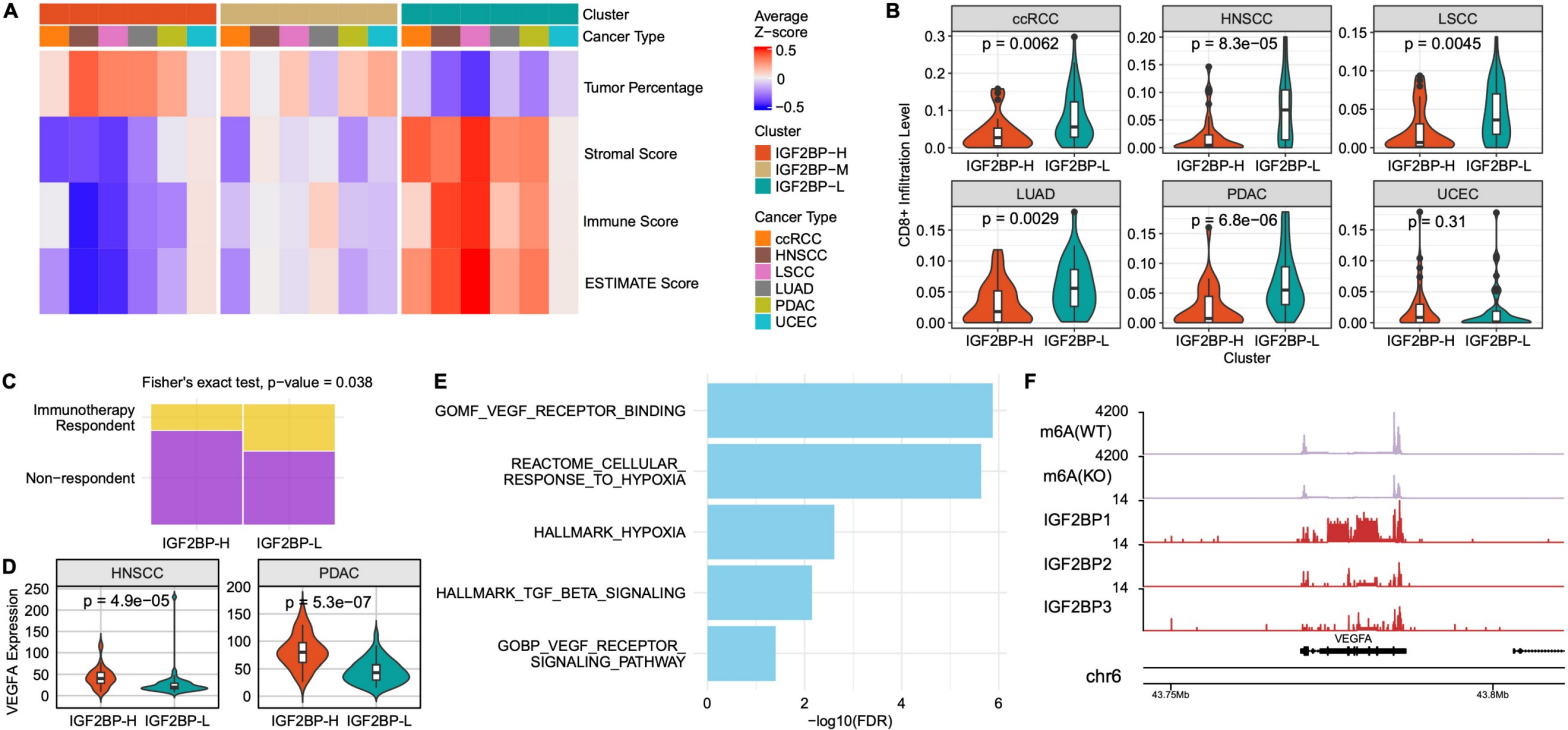
** Immune activity of IGF2BPs group**. **A:** Heatmap showing the average z-scores of tumor percentage, stromal score, immune score, and ESTIMATE score across the IGF2BP-H, -M, and -L groups for different cancer types. **B:** Violin plots comparing CD8^+^ T cell infiltration levels between the IGF2BP-H and -L groups across the six cancer types. Infiltration was significantly lower in the IGF2BP-H for most cancers. *P*-values were calculated using the Wilcoxon rank-sum test. **C:** Fisher's exact test was used to compare immunotherapy responses between the groups. The proportion of responders was significantly lower in the IGF2BP-H group than that in the IGF2BP-L group (*p* = 0.038). **D**: Violin plots comparing VEGFA expression levels between the IGF2BP-H and IGF2BP-L groups in HNSCC and PDAC. *P*-values were calculated using the Wilcoxon rank-sum test. **E**: Bar plot showing the enriched pathways in the IGF2BP-H PDAC group. The pathways related to hypoxia and angiogenesis were significantly upregulated in the IGF2BP-H group. **F**: Coverage plot displaying m6A-seq and RIP-seq data for the VEGFA transcript in HepG2 cells. m6A(WT) and m6A(KO) indicate m6A-seq coverage in wild-type and m6A writer knockout conditions. IGF2BP1, IGF2BP2, and IGF2BP tracks show RIP-seq coverage of each respective IGF2BP protein. The VEGFA transcript exhibits m6A methylation and IGF2BPs binding, suggesting post-transcriptional regulation by m6A readers. WT wild type, KO knock out.

Because the IGF2BP-H group exhibited a downregulated immune response pathway, we sought to characterize the immune landscape of this group. The IGF2BP-H group was characterized by a higher percentage of tumor cells and lower stromal, immune, and ESTIMATE scores, suggesting that immune cell infiltration was suppressed in this group (**Fig. 5A**). This indicated that the IGF2BP-H group was in a state of immune suppression or evasion, potentially facilitating tumor growth and progression. Consistent with this, a comprehensive analysis of individual cell infiltration levels revealed that immune cell infiltration was generally lower in the IGF2BP-H group (**Fig. S4A**). Specifically, the infiltration levels of CD8^+^ T cells were significantly lower in the IGF2BP-H group than in the IGF2BP-L group (*p* < 0.0045), except in UCEC (**Fig. 5B**). In UCEC, the overall immune cell infiltration rate was low, which may explain the lack of significant differences in this subgroup.

Based on these findings, we hypothesized that the IGF2BP-H group would exhibit poor response to immunotherapy. When predicting the response to immunotherapy in LSCC and LUAD, a significantly higher number of samples in the IGF2BP-H group were predicted to not respond to immune treatment (*p* = 0.038, Fisher's exact test), reinforcing the hypothesis that immune evasion mechanisms are involved (**Fig. 5C**). This highlights the need for alternative therapeutic strategies as standard immune checkpoint inhibitors may be less effective in these patients.

The suppressed immune response observed in the IGF2BP-H group prompted further investigation of potential immunosuppressive mechanisms. By examining the expression levels of immunosuppressive cytokines in the IGF2BP-H and IGF2BP-L groups, we found that VEGFA expression was significantly higher in the IGF2BP-H group in both HNSCC and PDAC (**Fig. 5D**). VEGFA is a well-known cytokine that promotes angiogenesis and vascular permeability and has been implicated in creating an immunosuppressive tumor microenvironment by inhibiting the function of immune cells, such as T cells and dendritic cells 61. Notably, in the PDAC subgroup, the IGF2BP-H group exhibited significantly elevated enrichment scores associated with angiogenesis and hypoxia, suggesting that these microenvironmental conditions may have contributed to immune suppression (**Fig. 5E**). Furthermore, VEGFA was identified as both a target of IGF2BPs and an m6A methylation site (**Fig. 5F**). In addition, HOXB9 was significantly upregulated in the high-expression group across multiple cancer types (**Fig. S4B**). Given its known roles in tumor progression and immune modulation 62, 63, HOXB9 upregulation may further contribute to the immune suppression observed in the IGF2BP-H group. These findings combined indicated that the overexpression of VEGFA in IGF2BP-H tumors, potentially regulated by IGF2BPs, may influence the immune microenvironment in HNSCC and PDAC, thereby contributing to the complex mechanisms underlying immune evasion.

## Discussion

The dynamic regulation of m6A within cells has been increasingly recognized in cancer biology, revealing complex patterns across various cancer types. In this study, we aimed to comprehensively elucidate the impact of m6A regulation and associated genes in defining cancer subgroups across multiple cancer types.

Our subgrouping analysis based on 25 m6A-associated genes revealed distinct m6A regulation-driven subgroups in six cancers, with IGF2BP family members (IGF2BP1, IGF2BP2, IGF2BP3; all known m6A readers) emerging as a central factor distinguishing these subgroups. Previous studies have established that IGF2BP proteins exert oncogenic effects by m6A-dependent binding to various oncogenic transcripts 39, 64. The frequent overexpression of IGF2BPs observed in cancer patients 65, along with substantial variation in expression levels between patients, likely explains their significant role in subgroup differentiation.

Clinically, samples with high IGF2BPs expression demonstrated poorer survival outcomes, particularly in ccRCC and LUAD. Notably, IGF2BPs overexpression in HNSCC and LSCC appeared primarily driven by CNVs, whereas in ccRCC, LUAD, PDAC, and UCEC, transcriptional activation of IGF2BP genes was predominant. Li et al. 66 previously reported that HNSCC and LSCC samples often belong to a multi-omic subgroup characterized by a high burden of copy number-altered oncogenes, potentially explaining the observed CNV-driven IGF2BPs overexpression in these cancers.

Existing research indicates that m6A modifications are pivotal in cancer progression and cell cycle regulation 67-69. Additional studies have shown that IGF2BPs enhance the expression of cell cycle-related genes through m6A-dependent mechanisms 39. In the present study, we further clarified how IGF2BPs regulate the cell cycle in various types of cancers. We observed that tumors exhibiting high IGF2BPs expression (IGF2BP-H) had significantly elevated cell cycle activity. Mechanistically, IGF2BPs bind to m6A sites on target genes such as TOP2A, ANLN, and TFRC, upregulating their expression and thereby driving cell cycle progression. These findings provide a molecular basis for the increased proliferative capacity seen in IGF2BP-H tumors.

The role of m6A in tumor immunity has also been the focus of recent research. m6A modifications influence both innate and adaptive immune responses, with studies implicating m6A modifications in genes, such as STAT1^70^ and FOXO3^71^ in immune cell activation. Our study adds to this body of knowledge by demonstrating that high IGF2BPs expression is correlated with suppressed immune responses in tumors. Specifically, IGF2BP-H tumors exhibited reduced immune cell infiltration, particularly of CD8^+^ T cells, which are critical for antitumor immunity. In PDAC and HNSCC, we observed that m6A-IGF2BPs-dependent upregulation of VEGFA was associated with immune suppression. These findings are consistent with previous research showing that IGF2BP1 knockout inflamed the tumor microenvironment by increasing NK cells and tumor-associated myeloid cells 45 and that high IGF2BP1 and IGF2BP3 levels are associated with immunotherapy resistance in melanoma patients 45.

Our study highlights the significant implications of m6A modifications in defining tumor subgroups and influencing cancer progression through post-transcriptional regulatory networks. Identifying IGF2BPs as a key driver of these networks suggested that targeting IGF2BPs interactions with oncogenic transcripts may represent a promising therapeutic strategy, particularly in the context of RNA-targeted therapies. However, our study has several limitations. Although we utilized multi-omics data to identify IGF2BPs-driven regulatory networks, further experimental validation across different cancer models is necessary to confirm the functional impact of these interactions. Additionally, the broader landscape of RNA modifications, including m5C and pseudo-uridylation, warrants further investigation to fully understand the epitranscriptomic regulation of cancer cells.

In conclusion, our findings provide new insights into the roles of m6A and IGF2BPs in cancer biology, particularly in regulating cell cycle activity and immune responses. By defining m6A-driven subgroups and elucidating the mechanisms underlying IGF2BP-mediated oncogenesis, this study contributes to the growing understanding of epitranscriptomic regulation in cancer and highlights potential avenues for therapeutic interventions. Future studies should focus on validating these findings using experimental models and exploring the interplay between m6A and other RNA modifications to elucidate the complexities of cancer biology further.

## Supplementary Material

Supplementary figures and tables.

## Abbreviations

m6A: N^6^-methyladenosine
BRCA: Breast cancer
ccRCC: Clear cell renal cell carcinoma
COAD: Colon adenocarcinoma
GBM: Glioblastoma
HGSC: High-grade serous carcinoma
HNSCC: Head and neck squamous cell carcinoma
LUAD: Lung adenocarcinoma
LSCC: Lung squamous cell carcinoma
PDAC: Pancreatic ductal adenocarcinoma
UCEC: Uterine corpus endometrial carcinoma
IGF2BP-H: IGF2BP-High
IGF2BP-M: IGF2BP-Middle
IGF2BP-L: IGF2BP-Low
CNV: Copy number variation
TF: Transcription factor
FDR: False discovery rate
Log2FC: Log2 fold change
CDKs: Cyclin-dependent kinases
