# Supplementary material for: Proteogenomic Analysis on RNA m6A Modification-Associated Genes Identifies a Distinct Subgroup with High IGF2BPs Expression Across Cancer Types
Source: Int J Med Sci. 2025 Aug 16;22(15):3815–27. doi: 10.7150/ijms.115609 (PMC12492183; doi:10.7150/ijms.115609)
Supplement: Supplementary file 1 — Supplementary figures and tables. [file ijmsv22p3815s1.zip › Supplementary_Figures.pdf]

Supplemental Tables

Table S1 – (A) Normalized TPM matrix of 25 m6A associated genes. (B) Cluster information of samples in 6 cancer types (ccRCC, HNSCC, LSCC, LUAD, PDAC, UCEC). (C) Average expression levels of IGF2BP1,2 and 3 across subgroups of each cancer type.

Table S2 – (A) Log2 fold change of m6A associated gene expression between tumor and normal tissue. (B) Fisher's test results of copy number amplification comparisons. (C) Transcription factor activation analysis results.

Table S3 – (A) Gene set enrichment analysis result. (B) t-test results of proteomics data. (C) t-test results of phosphoproteomics data. (D) Protein-protein interaction enrichment analysis with metascape result. (E) t-test result of enriched pathways in each cancer and data type (RNA, protein, phosphoprotein).

Table S4 – (A) Differentially expressed protein analysis result of TOP2A, ANLN, and TFRC. (B) Differentially expressed gene analysis result of TOP2A, ANLN and TFRC when silencing IGF2BP1/2/3. (C) Differentially expressed analysis result of TOP2A, ANLN, and TFRC when silencing METTL3. (D) Activated kinases in IGF2BP-H group in each cancer type. (E) Enriched pathways in samples with high expression of TOP2A, ANLN and TFRC.

Table S5 – (A) Immunotherapy response prediction of LSCC, LUAD samples with TIDE.

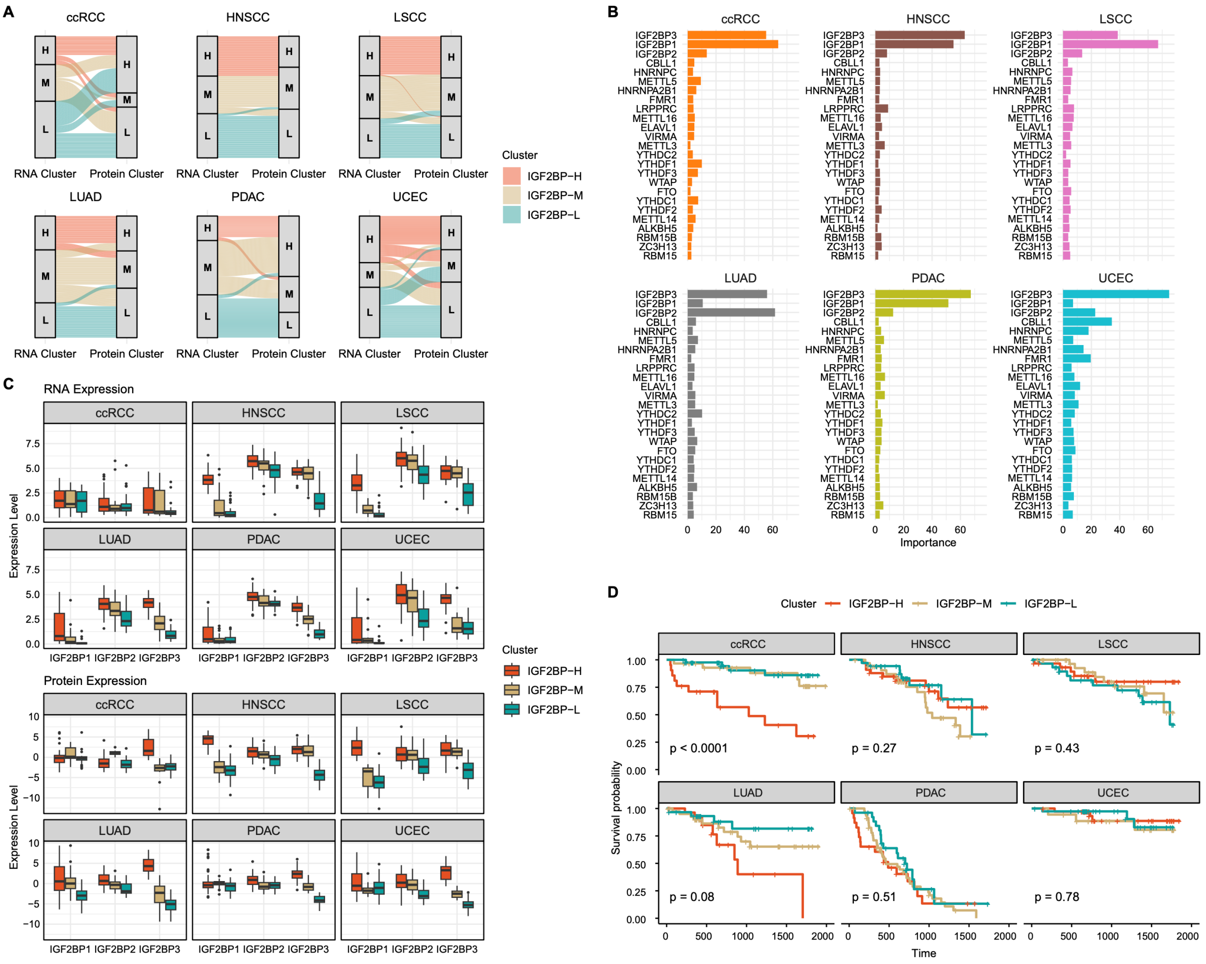

Figure S1. A. Alluvial plot illustrating the comparison between transcriptomics-based and proteomics-based clustering in multiple cancer types (ccRCC, HNSCC, LSCC, LUAD, PDAC, and UCEC). **B.** Feature importance analysis of 25 m6A regulatory genes, ranked by their average contribution to clustering across different cancers. IGF2BP family genes show the highest importance in all cancer types. **C.** Box plots comparing IGF2BP1/2/3 expression levels in RNA and protein datasets within the proteomics-based clusters. **D.** Kaplan-Meier survival curves for 6 cancer types, comparing overall survival between IGF2BP-H, IGF2BP-M and IGF2BP-L groups.

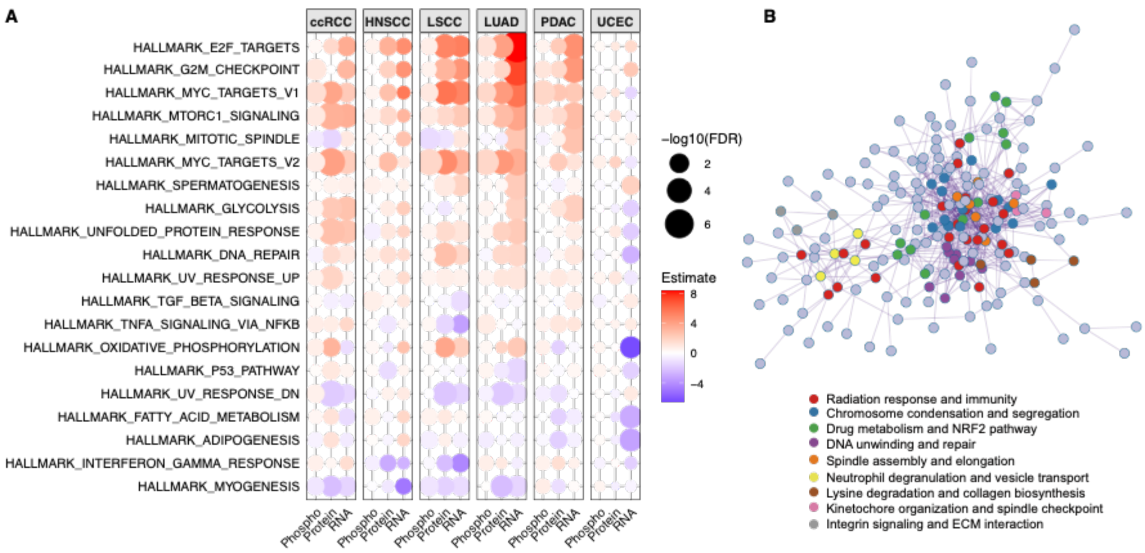

Figure S2. A. Bubble plot comparing IGF2BP-H and IGF2BP-L across transcriptome, proteome, and phosphoproteome data in six cancer types. The color gradient represents the estimate value, while the bubble size corresponds to the statistical significance (-log10 FDR). **B**. Protein-protein interaction (PPI) enrichment analysis results. Each color represents densely connected network components, functionally described by the most representative pathway associated with the constituent proteins.

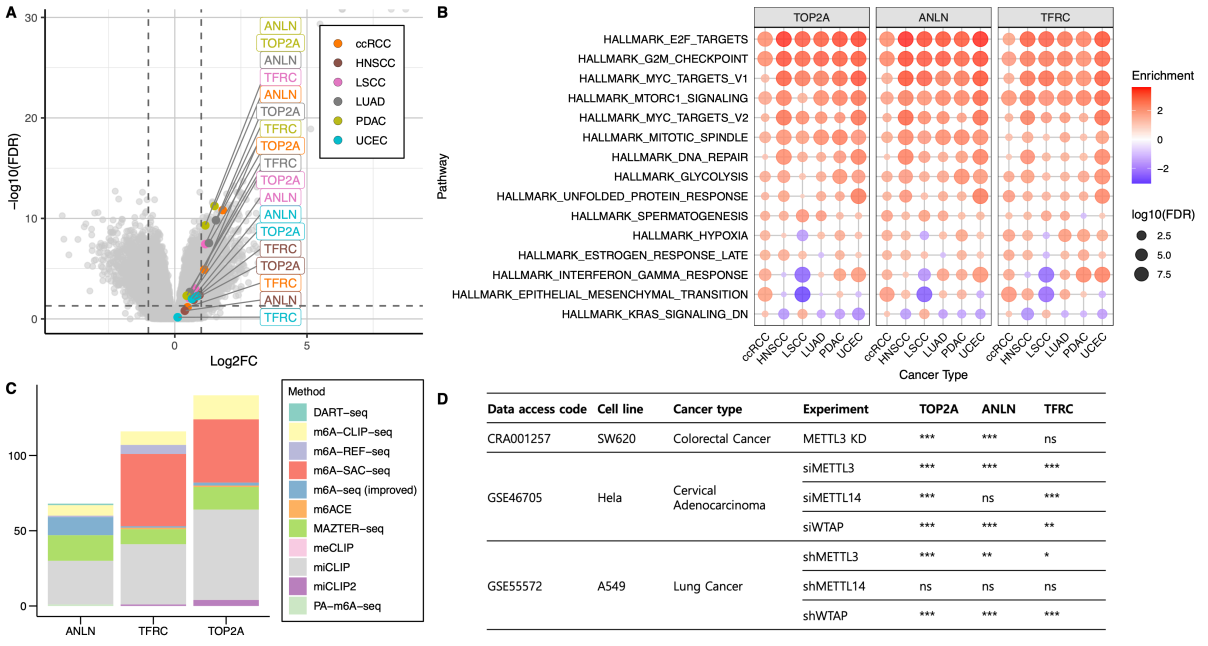

Data access code	Cell line	Cancer type	Experiment	TOP2A	ANLN	TFRC
CRA001257	SW620	Colorectal Cancer	METTL3 KD	***	***	ns
GSE46705	Hela	Cervical Adenocarcinoma	siMETTL3	***	***	***
			siMETTL14	***	ns	***
			siWTAP	***	***	**
GSE55572	A549	Lung Cancer	shMETTL3	***	**	*
			shMETTL14	ns	ns	ns
			shWTAP	***	***	***

Figure S3. A. Volcano plot showing the expression levels of IGF2BP cell cycle-related target genes (TOP2A, ANLN, and TFRC) in 6 cancer types at the transcriptome level. **B**. Pathways enriched in patient samples from six cancer types when grouped by high and low expression levels of TOP2A, ANLN, and TFRC. The bubble size represents statistical significance (-log10 FDR), and the color indicates enrichment score (NES). **C.** Summary of m6A peak occurrences on the indicated genes (ANLN, TFRC, and TOP2A) compiled from the m6A-Atlas database. The y-axis represents the total number of reported m6A peaks per gene aggregated across all methods. **D.** Summary of differential gene expression (DEG) analyses performed using publicly available m6A-seq datasets under various m6A writer knockdown or knockout conditions (METTL3, METTL14, WTAP). Each row corresponds to the result of a DEG test performed in a specific cell line and experimental condition. The significance annotations in the gene columns (TOP2A, ANLN, and TFRC) indicate that the respective gene was significantly downregulated under the indicated condition. *FDR < 0.05, **FDR < 0.01, ***FDR < 0.001; ‘ns’ indicates not significant.

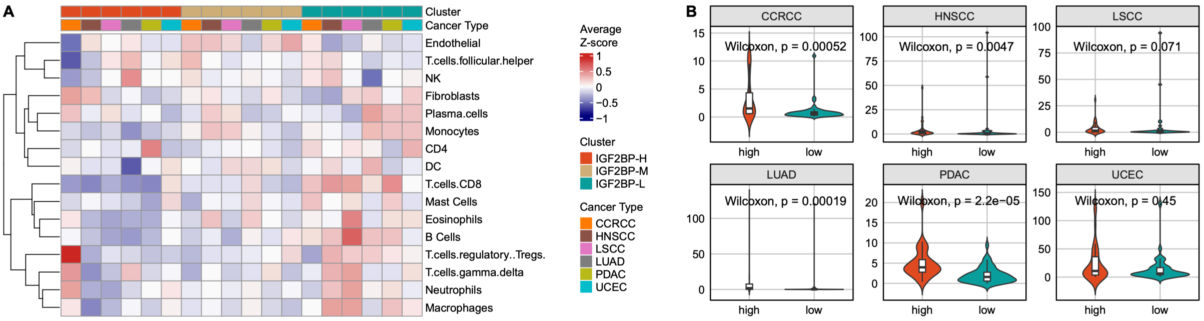

Figure S4. A. Heatmap displaying the average infiltration levels of various immune and stromal cell types across multiple cancer types. The infiltration scores are normalized within each cancer type and then averaged for each cluster. **B**. Violin plots depicting the distribution of HOXB9 gene expression levels between IGF2BP-H and IGF2BP-L groups across different cancer types. The Wilcoxon rank-sum test was used to assess statistical significance.
